# Enhancement of Luminous Intensity Emission from Incoherent LED Light Sources within the Detection Angle of 10° Using Metalenses

**DOI:** 10.3390/nano12010153

**Published:** 2022-01-01

**Authors:** Hanlyun Cho, Heonyeong Jeong, Younghwan Yang, Trevon Badloe, Junsuk Rho

**Affiliations:** 1 Department of Mechanical Engineering, Pohang University of Science and Technology (POSTECH), Pohang 37673, Korea; hanlyun.cho@postech.ac.kr (H.C.); jhyng2@postech.ac.kr (H.J.); younghwan@postech.ac.kr (Y.Y.); trevon@postech.ac.kr (T.B.); 2 Department of Chemical Engineering, Pohang University of Science and Technology (POSTECH), Pohang 37673, Korea; 3 POSCO-POSTECH-RIST Convergence Research Center for Flat Optics and Metaphotonics, Pohang 37673, Korea

**Keywords:** metalens, nanohole meta-atom, light-emitting diode, incoherent light source, surface light source, far field propagation

## Abstract

In this work, we present metalenses (MLs) designed to enhance the luminous intensity of incoherent light-emitting diodes (LEDs) within the detection angles of 0° and 10°. The detection angle of 0° refers to the center of the LED. Because the light emitted from LEDs is incoherent and expressed as a surface light source, they are numerically described as a set of point sources and calculated using incoherent summation. The titanium dioxide (TiO_2_) and amorphous silicon (a-Si) nanohole meta-atoms are designed; however, the full 2π phase coverage is not reached. Nevertheless, because the phase modulation at the edge of the ML is important, an ML is successfully designed. The typical phase profile of the ML enhances the luminous intensity at the center, and the phase profile is modified to increase the luminous intensity in the target detection angle region. Far field simulations are conducted to calculate the luminous intensity after 25 m of propagation. We demonstrate an enhancement of the luminous intensity at the center by 8551% and 2115% using TiO_2_ and a-Si MLs, respectively. Meanwhile, the TiO_2_ and a-Si MLs with the modified phase profiles enhance the luminous intensity within the detection angle of 10° by 263% and 30%, respectively.

## 1. Introduction

Optical metasurfaces are made up of precisely designed structures, known as meta-atoms, that can modulate the phase [1,2], amplitude [3,4,5], and polarization [6,7] of incident light. Metalenses (MLs) [8,9,10,11,12,13,14], meta-holograms [13,14,15,16,17,18,19,20,21,22,23,24,25,26,27], structural color [28,29,30,31], light detection [32,33,34,35,36,37,38], perfect absorber [39,40], and sensors [41,42,43,44,45,46,47] are typical applications of optical metasurfaces. Recently, various improvements to MLs have been reported, such as achromatic MLs [48,49,50], polarization-insensitive MLs [50,51], wide field-of-view MLs [52,53], large area MLs [54,55], uses in augmented reality [55,56], tunable focal lengths [38,57,58,59], and fabrication using nanoimprint technology [60,61]. Because MLs are thin and light, they are a potential candidate to completely replace conventional optical lenses, and also have the ability to be combined with conventional bulk optical systems [62]. However, most reported MLs are designed for coherent light sources.

Light-emitting diodes (LEDs) are commonly used as practical light sources [63]. However, the luminous intensity from LEDs dramatically decreases as the light propagates due to the emission of diverging spherical waves [64]. The intensity of light extraction efficiency of LEDs has been enhanced using microlens arrays [65,66,67], surface roughening [68,69,70,71], photonic crystal patterning [72,73], patterned substrates [74,75], and surface plasmons [76,77,78]. However, metasurfaces including MLs are difficult to use for LED sources, as the emitted light is incoherent [79,80].

In this study, we numerically design an ML for LED sources. Because the light from the LED is incoherent and is expressed as a surface light source, the LED is described as a set of point sources and the numerical results are calculated using incoherent summation. The ML enhances the luminous intensity within the detection angles of 0° (center of the LED) and 10° by collimating the diverging light emitted from the LED. We target the detection angle of 10° by considering the trade-off between the wide field-of-view and long propagation distance [81]. The phase profile of the ML is modified to spread out the transmitted light to the target detection angle.

## 2. Results and Discussion

### 2.1. Light Source Design for Simulation

**Figure 1:**
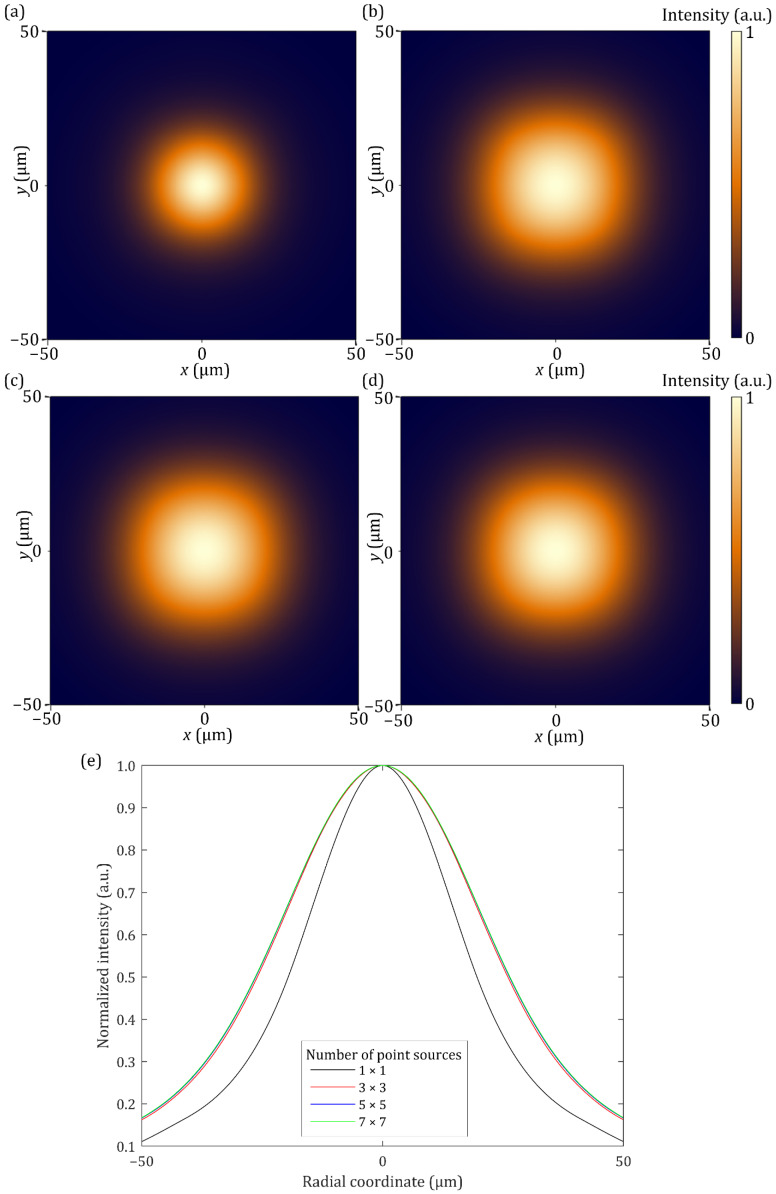
Simulated results after 20 μm of propagation with different numbers of points sources: (**a**) 1 × 1; (**b**) 3 × 3; (**c**) 5 × 5; (**d**) 7 × 7; (**e**) Intensity profiles. The results converge as the number of the point sources becomes 3 × 3.

The LEDs are described as numerous point sources with Lambertian intensity profiles that are incoherently summed. Thus, the designed LED source is an incoherent, unpolarized, surface light source. A 35 μm square LED source is used in this study. A micro-LED chip can be considered as a point source [82]. Figure 1 shows the simulated results after 20 μm of propagation with different numbers of points sources. The intensity profiles converge as the number of points sources become 3 × 3. In addition, the optimized and required chip size is at least 7 μm for typical display panels, including smartphones, laptops, and televisions [83,84,85], so 5 × 5 and 7 × 7 micro-chips are not considered in this study. We consider the LED source composed of 3 × 3 micro-LED chips, and the light source is described by 3 × 3 points sources. Because the light emitted from the LED is partially spatial-coherent, the phase of light originating from each point source is random and therefore interference effects are ignored. The propagation results shown in Figure 1 are obtained by adding only the amplitudes of the propagated light.

The Lambertian intensity profile is expressed as [86]

(1) $$I(\theta )=I_{0}\cos\theta$$

where *I* is the intensity, *θ* is the propagation angle with respect to the optical axis, and *I*_0_ is the maximum intensity at *θ* = 0, respectively. To describe the Lambertian point source, an interface between two different media is used (Figure S1). Because the transmittance at the interface is dependent on *θ*, the intensity of the transmitted light also varies with *θ*. When the distance between the point source and the interface is 40 nm and the refractive indices of the media are 1 and 2, the intensity profile of the transmitted light is similar to a Lambertian intensity profile, therefore allowing us to describe the single point source. The LED source is then modeled as 3 × 3 point sources placed 40 nm away from the interface. The schematics of the simulation is shown in Figure S2. Figure S3 shows the difference when a single point source and 3 × 3 points sources are used. However, individual simulations are done for each point source as the LED source is partially spatial-coherent, and therefore interference must be neglected [87]. The final calculations result from the incoherent summation of each individual simulation. The total number of simulations can be reduced if the system is symmetrically designed, allowing some results to be obtained by rotating other results. In addition, the partial temporal coherence of the LED source provides a broad spectral bandwidth of emitted light [88]. The simulation results with both narrowband and broadband sources provide similar results (Figure S4); therefore, the effect of the partial temporal coherence is negligible and ignored.

In this work, the points sources are placed at the center of the 3 × 3 lattice (Figure S2b). The effect of the relative locations of the sources is negligible in a 3 μm × 3 μm LED source, as the 3 × 3 array of dipole sources provides a similar light emission to a single point source of the same size [89].

### 2.2. Phase Profile of Metalens

**Figure 2:**
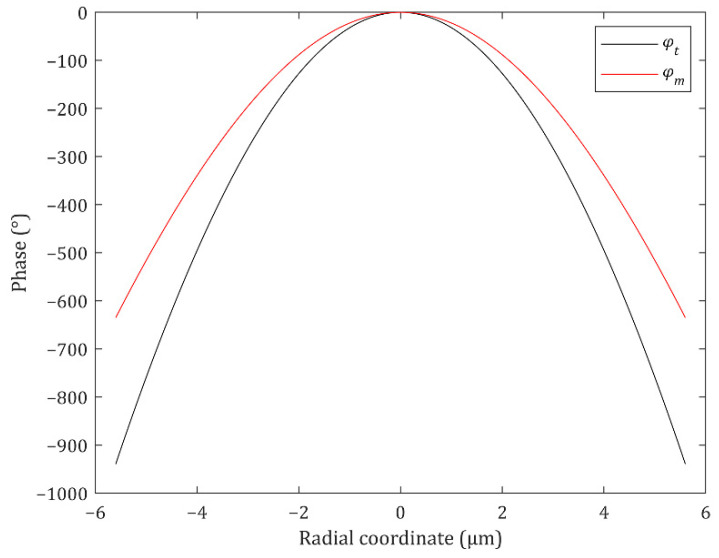
The typical (black line) and modified (red line) phase profiles of the metalens (ML) at *f* = 10 μm and *λ* = 560 nm.

The typical phase profile *φ_t_* of an ML is expressed as [50]

(2) $$\varphi_{t}(r,\lambda )=-\frac{2\pi }{\lambda }\left(\sqrt{r^{2}+f^{2}}-f\right)$$

where *r*, *λ*, and *f* are the radial coordinate, the wavelength, and the focal length, respectively. Spherical waves from the LED source are transmitted through the ML and collimated. However, the collimated light is concentrated at the propagation angle of 0°, so the phase profile is modified to enhance the luminous intensity within the detection angle of 10°. The modified phase profile *φ_m_* is expressed as

(3) $$\varphi_{m}(r,\lambda )=-\frac{2\pi }{\lambda }\left[\left(\sqrt{r^{2}+f^{2}}-f\right)-\left(\sqrt{r^{2}+{\left(\frac{L}{2m\tan\alpha }\right)}^{2}}-\frac{L}{2m\tan\alpha }\right)\right]$$

where *L* is the length of the ML, *m* is the number of point sources that make up of the LED source, and *α* is the detection angle. *L* is 35 μm, the same with the length of the LED source, *m* is 3 because 3 × 3 points sources are used, and *α* is 10° in this study. Because the lengths of the ML and the LED are identical, an ML array can be used to cover a large-area LED composed of equally sized LED pixels. The phase profile from Equation (3) is obtained by adding the phase profiles of the typical ML (Equation (2)) and a concave lens (Figure S5). The second term of Equation (3) originates from the concave lens allowing the transmitted light to spread out to the target detection angle region. Because the LED source is a surface light source and is described as a set of the points sources, the designed ML is split into the subsections one-to-one correspondence with the point sources and the phase profile in each subsection is defined by Equation (2) or (3). The phase profiles at *f* = 10 μm and *λ* = 560 nm are shown in Figure 2. The phase gradient of *φ_m_* becomes smaller at the edge of the ML than that of *φ_t_*. Thus, when *φ_m_* is used, the diverging light from the source is steered less and spreads out to the target detection angle region.

### 2.3. Nanohole Meta-Atom

Since LEDs emit unpolarized light, polarization-insensitive meta-atoms are designed. In addition, nanohole meta-atoms are used instead of nanofins due to good adhesion between the nanohole structure and the substrate when the high aspect ratio (above 1:5) nanostructures are fabricated (Figure S6). Because the dense material is continuously connected, the nanohole structure provides a high production yield. However, the nanofin structure can be relatively easily destroyed during the etching or cleaning process. When the optical axis of the system is parallel to the *z*-direction, the transmitted electric field through the nanohole is expressed as [50,90,91,92]

(4) $$\left[\begin{array}{c} E_{x}\\ E_{y} \end{array}\right]=\frac{t_{l}+t_{s}}{2}\left[\begin{array}{c} 1\\ \pm i \end{array}\right]+\frac{t_{l}-t_{s}}{2}\exp\left(\pm i2\beta \right)\left[\begin{array}{c} 1\\ \mp i \end{array}\right]$$

where *t*_l_ and *t_s_* are complex transmission coefficients when the incident light is polarized along the long and short axis of the nanohole, respectively, and *β* is the rotation angle of the nanohole with respect to the *x*-axis. The first and the second terms of Equation (4) are known as the co- and cross-polarization terms, respectively, because the Jones vector of each term shows that the polarization of the transmitted light is identical to the incident light at the first term and is opposite the incident light at the second term. The polarization dependence is caused by exp(±*i*2*β*) from the cross-polarization term. Therefore, the meta-atoms are designed to have the same *t*_l_ and *t_s_* to remove the cross-polarization term, which can be achieved using symmetrical meta-atoms such as circle and square holes (Figure S7).

**Figure 3:**
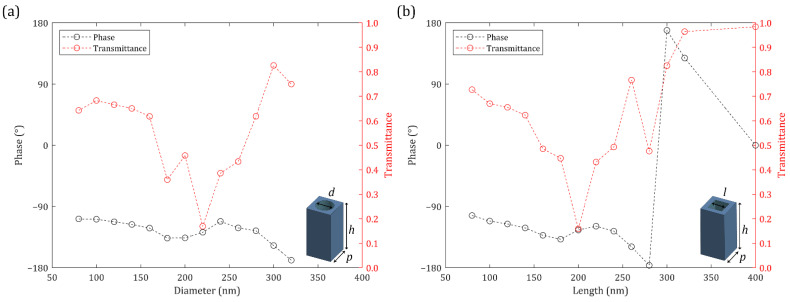
Transmission properties of Titanium dioxide (TiO_2_) meta-atoms at *λ* = 560 nm: (**a**) Circle nanohole meta-atoms; (**b**) Square nanohole meta-atoms. *p* = 400 nm and *h* = 400 nm.

**Figure 4:**
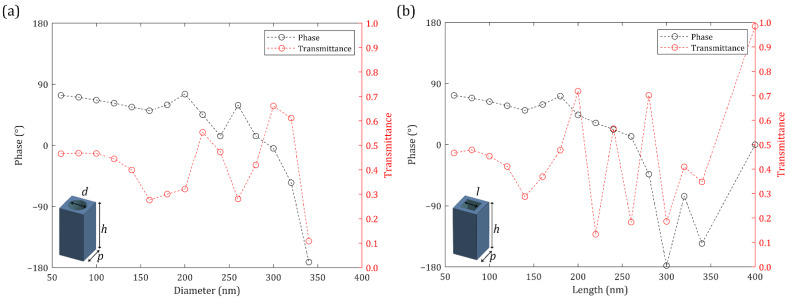
Transmission properties of amorphous silicon (a-Si) meta-atoms at *λ* = 560 nm: (**a**) Circle nanohole meta-atoms; (**b**) Square nanohole meta-atoms. *p* = 400 nm and *h* = 600 nm.

Titanium dioxide (TiO_2_) and amorphous silicon (a-Si) nanohole meta-atoms are usually used at visible frequencies due to their high refractive indices and low loss [93]. Gallium nitride (GaN) has also been used at visible frequencies; however, a sapphire substrate is required and the sapphire substrate is less transparent than fused silica [49]. Thus, TiO_2_ and a-Si are used in this study. The transmission properties of TiO_2_ nanohole meta-atoms with a height of 400 nm are investigated using rigorous coupled-wave analysis (RCWA) simulations (Figure 3) [94]. The designed meta-atoms work under the spherical wave incidence even though they are designed by RCWA under the plane wave incidence (Figure S8). The phase shown in Figure 3 does not satisfy the complete 2π coverage. However, they can be used to construct an ML even though the meta-atom library does not fully cover the phase from −π to π [54]. Meanwhile, the transmission properties of a-Si nanohole meta-atoms with height of 600 nm are also calculated using RCWA simulations (Figure 4). The fabrication process for a-Si meta atoms is relatively easier and is less restrictive than that of TiO_2_ meta-atoms, so a-Si meta-atoms can be designed with higher aspect ratios. Nevertheless, the phase depicted in Figure 4 does not cover the entire 2π range. The averaged transmittance of a-Si meta-atoms is lower than that of TiO_2_ meta-atoms.

**Figure 5:**
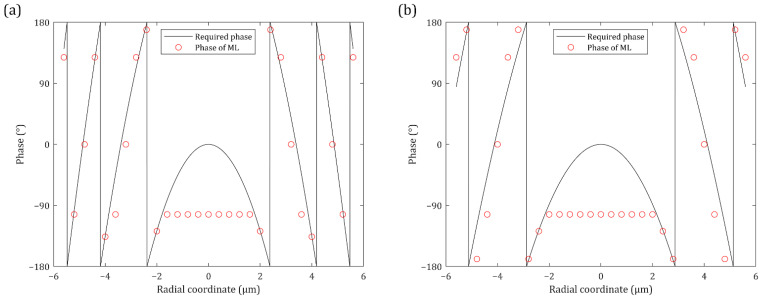
Comparisons of the required (black line) and the designed (red dot) phase of the MLs at *f* = 10 μm and *λ* = 560 nm: (**a**) The TiO_2_ ML with the typical phase profile; (**b**) The TiO_2_ ML with the modified phase profile.

**Figure 6:**
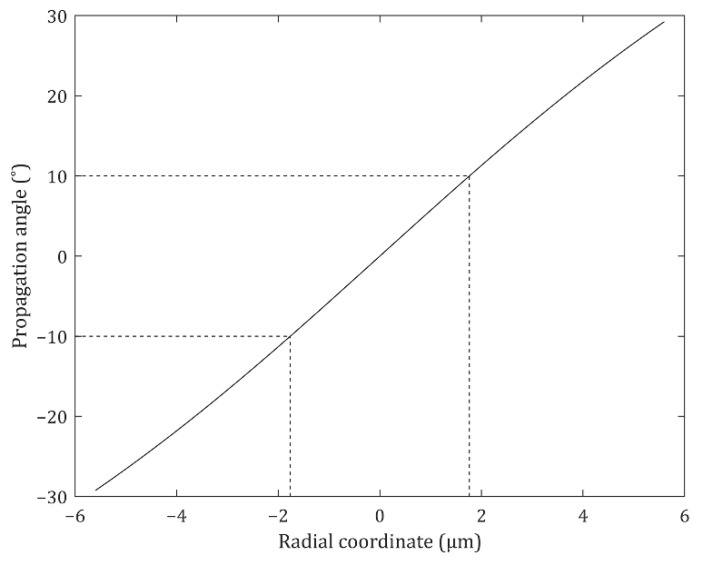
Propagation angle of the emitted light from the light-emitting diode (LED) source. Because the propagation angle at *r* < 1.76 μm is already below the target detection angle 10°, the phase modulation at the edge is enough to construct the MLs.

Figure 5 shows the phases of the designed TiO_2_ MLs. The phase profile at the center of the lens is somewhat different from the desired phases. However, the *θ* of light at the center is already below 10° (Figure 6) and is smaller than 10° at *r* < 1.76 μm. Thus, the transmitted light can reach the target detection angle region regardless of the zero phase gradient at the center. The MLs should steer the incident light at the edge to enhance the intensity within the target detection angle of 10°. Therefore, beam control at the edge is enough to construct the MLs, and the phase modulation at the edge is important for these designs.

### 2.4. Far Field Propagation Simulation

**Figure 7:**
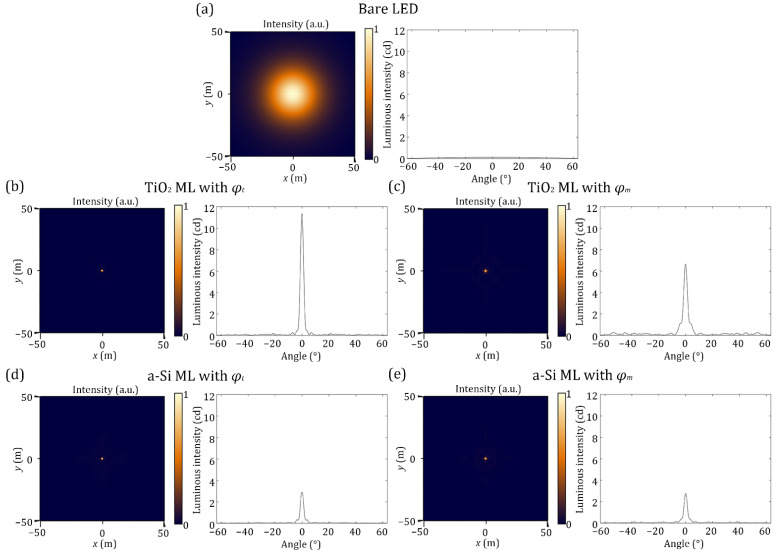
Simulated results after 25 m propagation with respect to the propagation angle *θ*: (**a**) The bare LED without any ML; (**b**) The TiO_2_ ML with the typical phase profile; (**c**) The TiO_2_ ML with the modified phase profile; (**d**) The a-Si ML with the typical phase profile; (**e**) The a-Si ML with the modified phase profile. The MLs with the modified phase profile provide lower but wider intensity profiles than those of the MLs with the typical phase profile.

The diverging spherical wave from the LED source passes through the ML and is able to propagate to the far field as the ML collimates the transmitted light. The emitted light from the LED source is at a distance *f* from the ML, then propagates for 25 m. The simulated propagation results are shown in Figure 7. The luminous intensities are calculated. Each ML enhances the luminous intensity at the center and the luminous intensity profile shows the peak near the angle of 0°. The peaks of the MLs with *φ_t_* at the center are higher but narrower than the MLs with *φ_m_* regardless of the materials. Because the cross-sectional intensity profiles shown in Figure 7 are radially symmetric, the intensity at large angles are more dominant to enhance the total luminous intensity. Table 1 shows the luminous intensities of the MLs with respect to the angle from 0° (center) to 10° (target detection angle). The MLs with *φ_m_* have larger luminous intensities, except at the angle of 0°, than that of the MLs with *φ_t_* for both materials. In addition, full widths at half maximum of the TiO_2_ MLs with *φ_t_* and *φ_m_* after 25 m of propagation are 1.22 and 1.45 m, respectively, and the full widths at half maximum of the a-Si MLs with *φ_t_* and *φ_m_* are 1.24, and 1.26 m, respectively.

**Figure 8:**
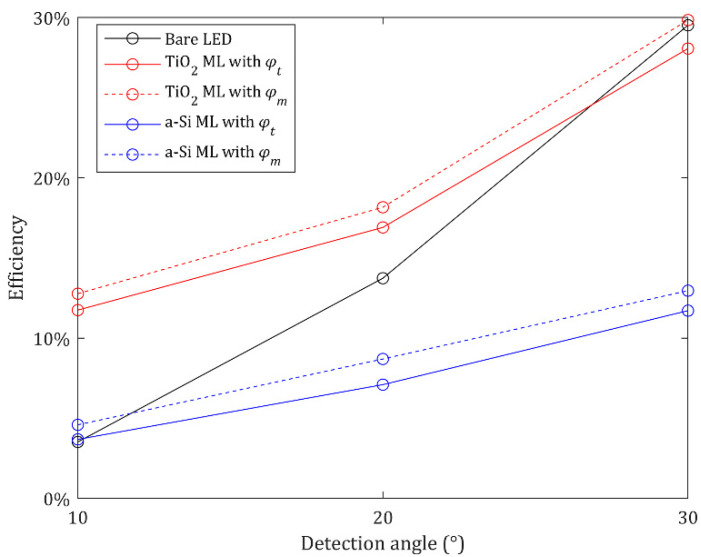
Efficiencies of the LED source and MLs within the different detection angles *α*.

The MLs enhance the peak intensity by steering the direction of the transmitted light but do not increase the light extraction efficiency of the LED. In addition, some portion of light is reflected or absorbed by the MLs. Therefore, the enhancement of the luminous intensity at the center implies that the intensity at the other areas decreases. The efficiencies of the MLs are shown in Figure 8 with respect to *α*. The efficiency is defined as the output luminous intensity within *α* (Figure S9) divided by the luminous intensity of the LED source. The TiO_2_ ML with *φ_m_* has higher efficiencies within *α* = 10°, 20°, and 30° than those of the bare LED, but the ML with *φ_t_* has lower efficiency within *α* = 30°. The luminous intensity within *α* = 10° is enhanced by 234% and 263% compared with the LED source when the TiO_2_ MLs with *φ_t_* and *φ_m_* are used, respectively (Table S1). Because the ML with *φ_m_* has the wide intensity peak profile, the ML has a higher luminous intensity within *α* = 10° than that of the ML with *φ_t_* despite the lower maximum luminous intensity at the center. Meanwhile, the enhancement of the luminous intensity within *α* = 20° and 30° is not significant, because the enhancement within *α* = 10° is a consequence of steering the transmitted light from the other area to the target detection angle region. The a-Si ML with *φ_m_* provides the higher efficiencies within *α* = 10°, 20°, and 30° than those of the a-Si ML with *φ_t_* due to the wider intensity profile. However, the a-Si MLs enhance the efficiencies slightly within *α* = 10° and have lower efficiencies within *α* = 20° and 30° due to the low transmittance of a-Si meta-atoms.

## 3. Conclusions

The MLs are designed to enhance the luminous intensity of incoherent LED sources after 25 m of propagation within *α* = 0° and 10°. The LED source is composed of 3 × 3 micro-chips and is designed as the set of 3 × 3 Lambertian point sources. The propagation results are obtained by incoherently adding the simulation result with each single point source. Polarization-insensitive nanohole meta-atoms are designed. Although the phase profiles of the designed MLs do not match with the required phases at the center, the emitted light from the LED source at the center already propagates in the target detection angle region, so the phase modulation at the edge of the MLs is important in this study. When the TiO_2_ and a-Si MLs with *φ_t_* are used, the luminous intensity at the center is enhanced by 8551% and 2115%, respectively. Meanwhile, the TiO_2_ and a-Si MLs with *φ_m_* enhance the luminous intensity within *α* = 10° by 263% and 30%, respectively. Because the TiO_2_ meta-atoms have higher transmittance than that of a-Si, the TiO_2_ ML shows the higher enhancement. Meanwhile, the efficiency enhancement within *α* = 20° or 30° is not significant because the ML cannot increase the light extraction efficiency of the LED source and the enhancement within *α* = 0° or 10° is a consequence of steering the transmitted light. Therefore, the emitted light from the LED source can be delivered over a long distance using the ML, proving that it can be employed for various practical applications using LEDs, including the automotive headlights, display panels and so on.

## 4. Methods

Commercially available Lumerical FDTD and VirtualLab Fusion were used for the far field simulations. The electric field transmitted through the ML was calculated using Lumerical FDTD. Then the electric field data were imported to VirtualLab Fusion and used as the source for the far field propagation. Finally, the simulated far field propagation results from each individual point source were incoherently summed up.

## Figures and Tables

**Table 1:** Luminous intensity of MLs at different propagation angles *θ*. Unit: cd.

	Propagation Angle (*θ*)				
	0°	2.5°	5°	7.5°	10°
Without ML (bare LED)	0.1315	0.1301	0.1297	0.1290	0.1271
TiO_2_ ML with *φ_t_*	11.37	1.107	0.1228	0.2157	0.05475
TiO_2_ ML with *φ_m_*	6.668	1.562	0.7678	0.1265	0.1720
a-Si ML with *φ_t_*	2.912	0.3451	0.1022	0.06574	0.08123
a-Si ML with *φ_m_*	2.778	0.4787	0.1306	0.1454	0.03788

## Data Availability

The data presented in this study are available on request from the corresponding author.

## Supplementary Materials

The following are available online at https://www.mdpi.com/article/10.3390/nano12010153/s1, Figure S1: Description of a point source with Lambertian intensity profile: (a) Schematics; (b) The Lambertian (black line) and simulated (red line) intensity profiles; Figure S2: Schematics of the simulation in this work: (a) The simulation from the LED source to the monitor is carried out using Lumerical FDTD. The far field simulation from the monitor to the detector is conducted using VirtualLab Fusion; (b) Distribution of the 3 × 3 points sources; Figure S3: Comparisons of the simulated results calculated from a single point source and 3 × 3 points sources; Figure S4: Comparisons of the simulated results with a narrowband source at *λ* = 560 nm and broadband source at 500 ≤ *λ* ≤ 620 nm; Figure S5: Description of the effect of the ML with *φ_m_* explained by geometrical optics; Figure S6: Good adhesion of nanohole meta-atoms: (a) a-Si nanostructure composed of nanofin meta-atoms; (b) a-Si nanostructure composed of nanohole meta-atoms. Scale bar: 1 μm; Figure S7. Comparison of *t*_l_ (solid line) and *t*_s_ (circle dot) of TiO_2_ meta-atoms: (a) Circle nanohole meta-atoms; (b) Square nanohole meta-atoms; Figure S8: Simulation result of the collimation using the TiO_2_ ML with *φ_t_*. The focal length is 3 μm. The phase is plotted instead of the intensity, because the intensity at the point source is extremely larger than that at the other area; Figure S9: Measurement of the intensity within a detection angle of *α*. *α* = 0° or 10° and *l* = 25 m; Table S1: Comparison of enhancement of intensity from LED sources of the previously reported results.
