# Supplementary material for: Enhancement of Luminous Intensity Emission from Incoherent LED Light Sources within the Detection Angle of 10° Using Metalenses
Source: Nanomaterials (Basel). 2022 Jan 1;12(1):153. doi: 10.3390/nano12010153 (PMC8746482; doi:10.3390/nano12010153)
Supplement: Supplementary file 1 [file nanomaterials-12-00153-s001.zip › nanomaterials-1468637-supplementary materials.pdf]

Supplementary Materials for

Enhancement of Luminous Intensity Emission from Incoherent LED Light Sources within the Detection Angle of 10° using Metalenses

Hanlyun Cho [1], Heonyeong Jeong [1], Younghwan Yang [1], Trevon Badloe [1] and Junsuk Rho [1,2,3]*

[1] Department of Mechanical Engineering, Pohang University of Science and Technology (POSTECH), Pohang 37673, Republic of Korea; hanlyun.cho@postech.ac.kr (H.C.); jhyng2@postech.ac.kr (H.J.); younghwan@postech.ac.kr (Y.Y.); trevon@postech.ac.kr (T.B.)

[2] Department of Chemical Engineering Pohang University of Science and Technology (POSTECH), Pohang 37673, Republic of Korea

[3] POSCO-POSTECH-RIST Convergence Research Center for Flat Optics and Metaphotonics, Pohang 37673, Republic of Korea

* Correspondence: jsrho@postech.ac.kr

Simulation

The Lambertian intensity profile is mimicked based on an interface between two different media (Figure S1). The LED source depicted in Figure S2 is composed of 3 x 3 points sources, and each point source is identical to the description in Figure S1a. The distance from the interface shown in Figure S1a to the ML is 10 μm, so the point source is 10.04 μm away from the ML. FDTD simulation results using Lumerical FDTD are recorded by the monitor. Then, the recorded electric field data is imported to VirtualLab Fusion as light sources, and the far field simulation is calculated. The substrate consists of fused silica and the thickness of the substrate is 500 μm. Because the LED source is incoherent and the interference is neglected, only one point source is used for each simulation. Then, the complete result is merged using incoherent summation of the amplitudes of each simulation result. Therefore, the simulation should be iterative except the position of the point source, and the number of the iteration is the same as the number of the points sources. Because the system is symmetrically designed, the number of iterations can be reduced by rotating other results. Figure S3 shows the difference when a single point source and 3 x 3 points sources are used. The result from the single point source has the lower intensity profile. In addition, Figure S4 shows the simulated results from the narrowband source (λ = 560 nm) and broadband source with the bandwidth of 120 nm (500 ≤ λ ≤ 620 nm)are similar.

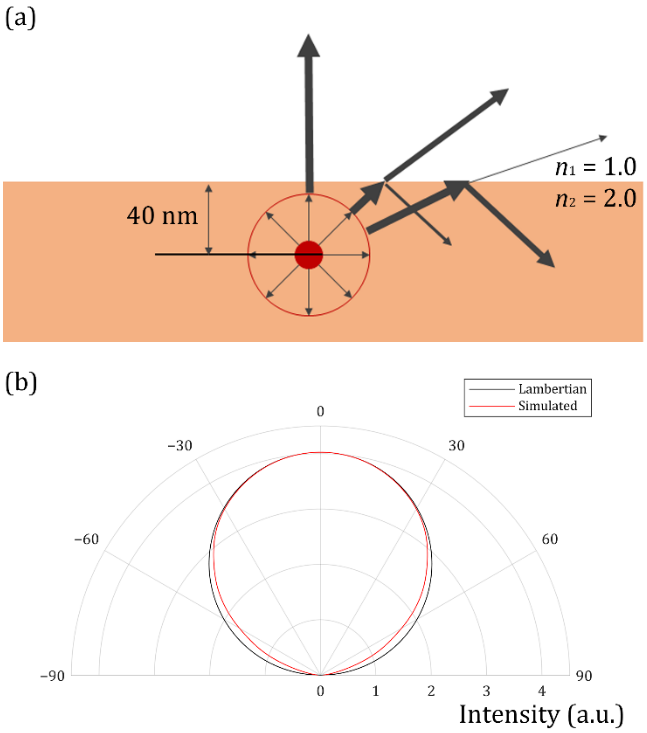

Figure S1. Description of a point source with Lambertian intensity profile: (a) Schematics; (b) The Lambertian (black line) and simulated (red line) intensity profiles.

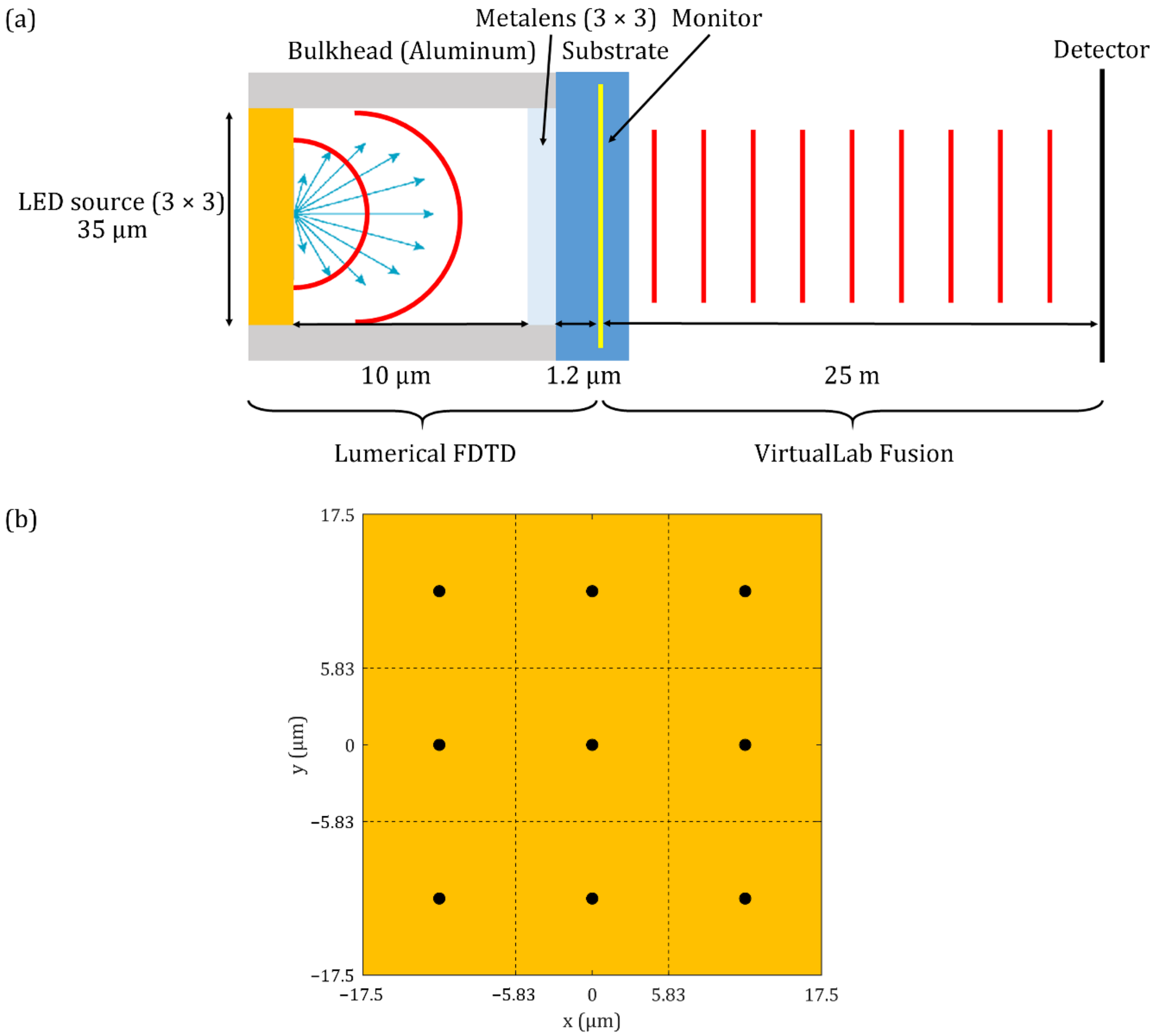

Figure S2. Schematics of the simulation in this work: (a) The simulation from the LED source to the monitor is carried out using Lumerical FDTD. The far field simulation from the monitor to the detector is conducted using VirtualLab Fusion; (b) Distribution of the 3 x 3 points sources.

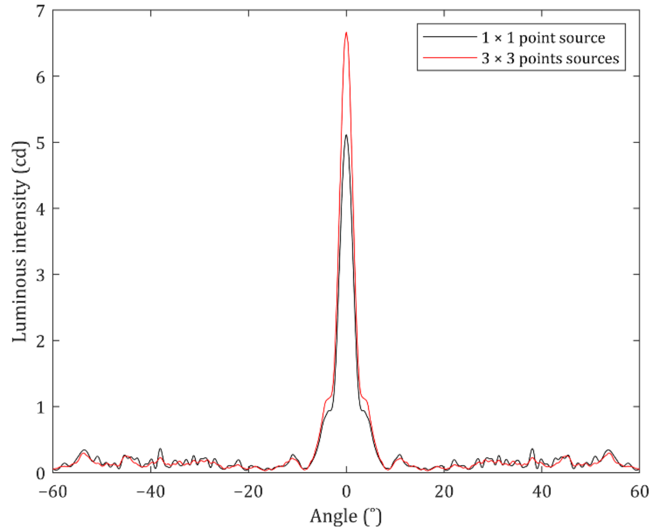

Figure S3. Comparisons of the simulated results calculated from a single point source and 3 x 3 points sources.

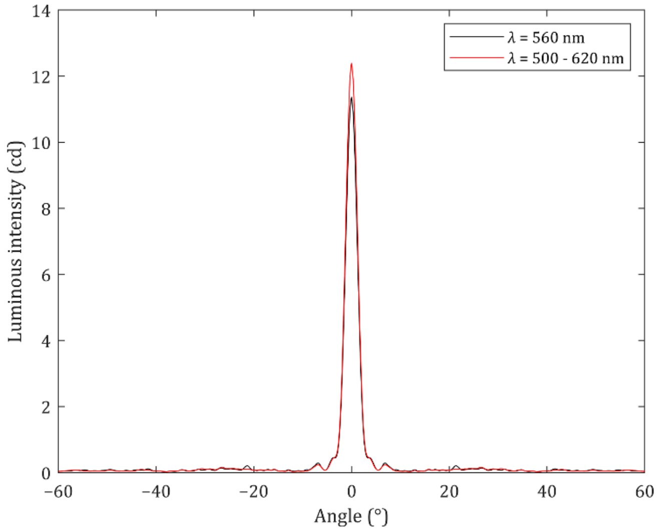

Figure S4. Comparisons of the simulated results with a narrowband source at λ = 560 nm and broadband source at 500 ≤ λ ≤ 620 nm.

Theoretical basis for Equation 3

The typical MLs with φ_t imitates only L1 shown in Figure S5, so the transmitted light is collimated. Meanwhile, the MLs with φ_m considers the effects of both L1 and L2 shown in Figure S5 to diverge the transmitted light to the target detection angle region. The description shown in Figure S5 considers each corresponding ML to the point source. The focal length of L2 f_{L2} varies depending on the size of the concave lens and the target detection angle, so f_{L2} is obtained by

$$f_{L2} = \frac{L}{2m\tan\alpha}. \tag{S1}$$

Therefore, the first and the second terms of Equation 3 originate from L1 and L2, respectively.

The gradients of φ_t and φ_m are expressed as

$$\frac{\partial\varphi_t}{\partial r} = -\frac{2\pi}{\lambda}\left(\frac{r}{\sqrt{r^2+f^2}}\right), \tag{S2}$$

$$\frac{\partial\varphi_m}{\partial r} = -\frac{2\pi}{\lambda}\left(\frac{r}{\sqrt{r^2+f^2}} - \frac{r}{\sqrt{r^2+f_{L2}^2}}\right), \tag{S3}$$

respectively. As r infinitely increases, the gradient of φ_t converges to $-2\pi/\lambda$ but the gradient of φ_m converges to 0. Thus, φ_m is not depicted as a hyperbolic curve unlike φ_t and not described as a phase profile of the typical ML with a different focal length.

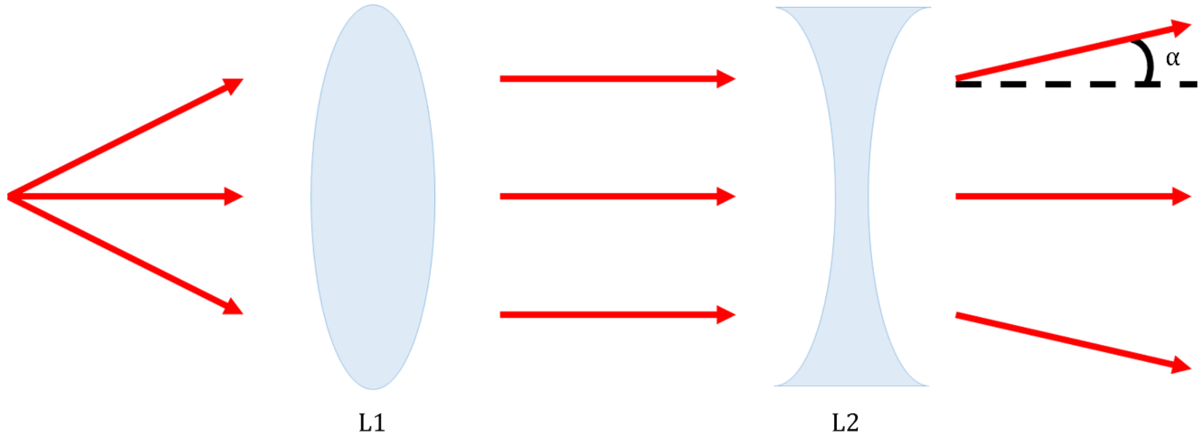

Figure S5. Description of the effect of the ML with φ_m explained by geometrical optics.

Good adhesion of nanohole meta-atoms

Nanohole structures provide better adhesion than those of nanofin structures (Figure S6) because the dense material is continuously connected. The nanostructures shown in Figure S6 are composed of a-Si and fabricated by electron-beam lithography.

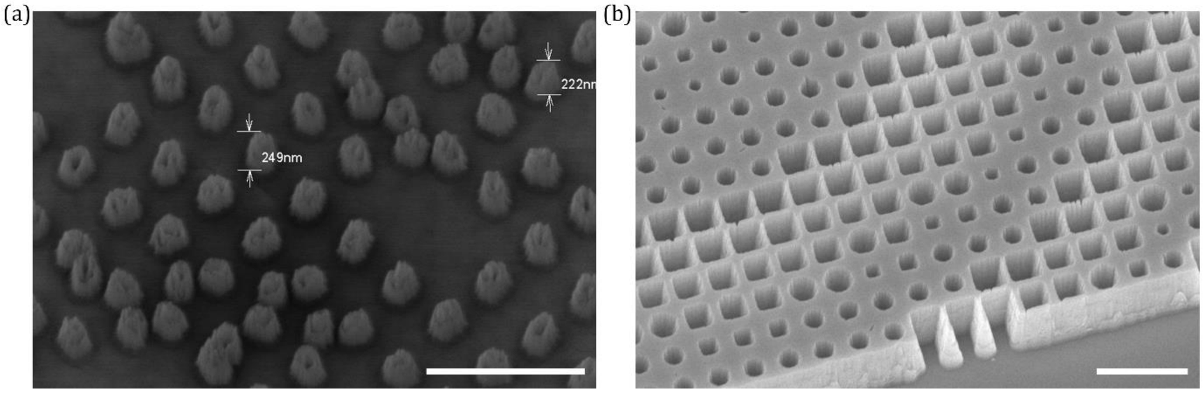

Figure S6. Good adhesion of nanohole meta-atoms: (a) a-Si nanostructure composed of nanofin meta-atoms; (b) a-Si nanostructure composed of nanohole meta-atoms. Scale bar: 1 μm.

Meta-atoms and RCWA simulation

Both circle and square nanohole meta-atoms provide the same t_l and t_s (Figure S7). The transmission properties are calculated by RCWA simulation with the TiO_2 meta-atoms with height of 400 nm. Thus, the ML composed of the circle or square meta-atoms is polarization insensitive. In addition, Figure S8 shows the TiO_2 ML with φ_t can collimate the spherical wave emitted from the point source well. The result is calculated by FDTD simulation and implies that the designed meta-atoms work under the spherical wave incidence even though they are designed by RCWA under the plane wave incidence.

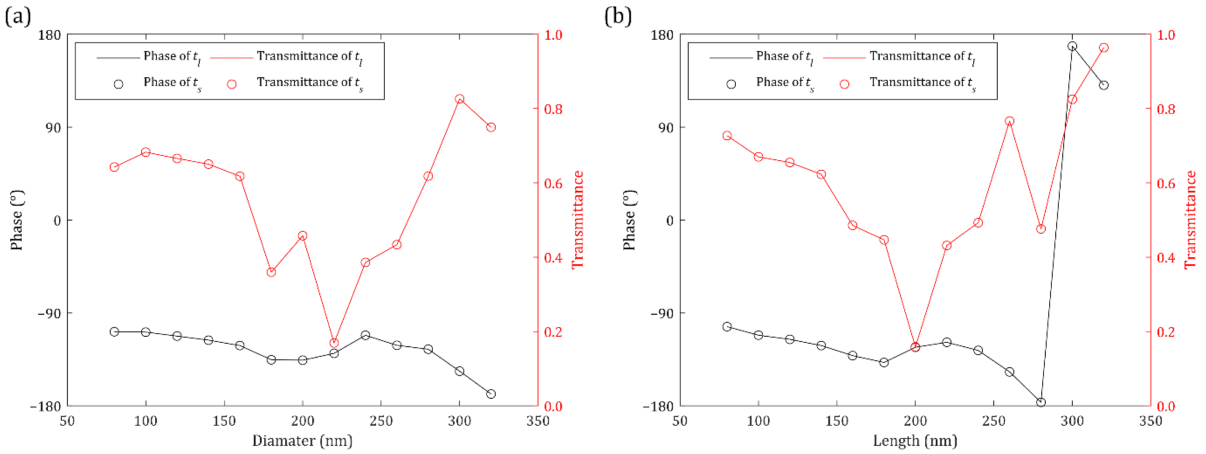

Figure S7. Comparison of t_l (solid line) and t_s (circle dot) of TiO_2 meta-atoms: (a) Circle nanohole meta-atoms; (b) Square nanohole meta-atoms.

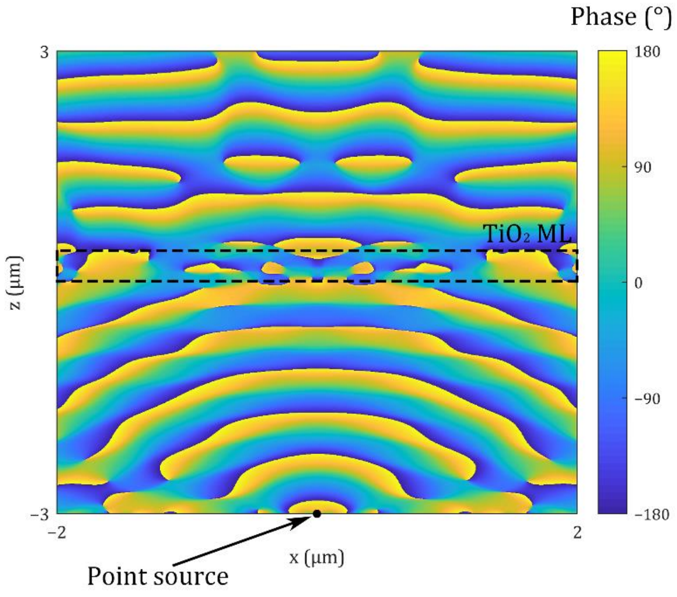

Figure S8. Simulation result of the collimation using the TiO_2 ML with φ_t. The focal length is 3 μm. The phase is plotted instead of the intensity, because the intensity at the point source is extremely larger than that at the other area.

Detection angle

The intensity within the detection angle of α is measured by the total intensity in the area where the propagation angle within α (Figure S9). Therefore, the intensity at $\alpha = 0°$ is the intensity at the center of the LED, and the intensity within $\alpha = 10°$ contains the intensity at the center.

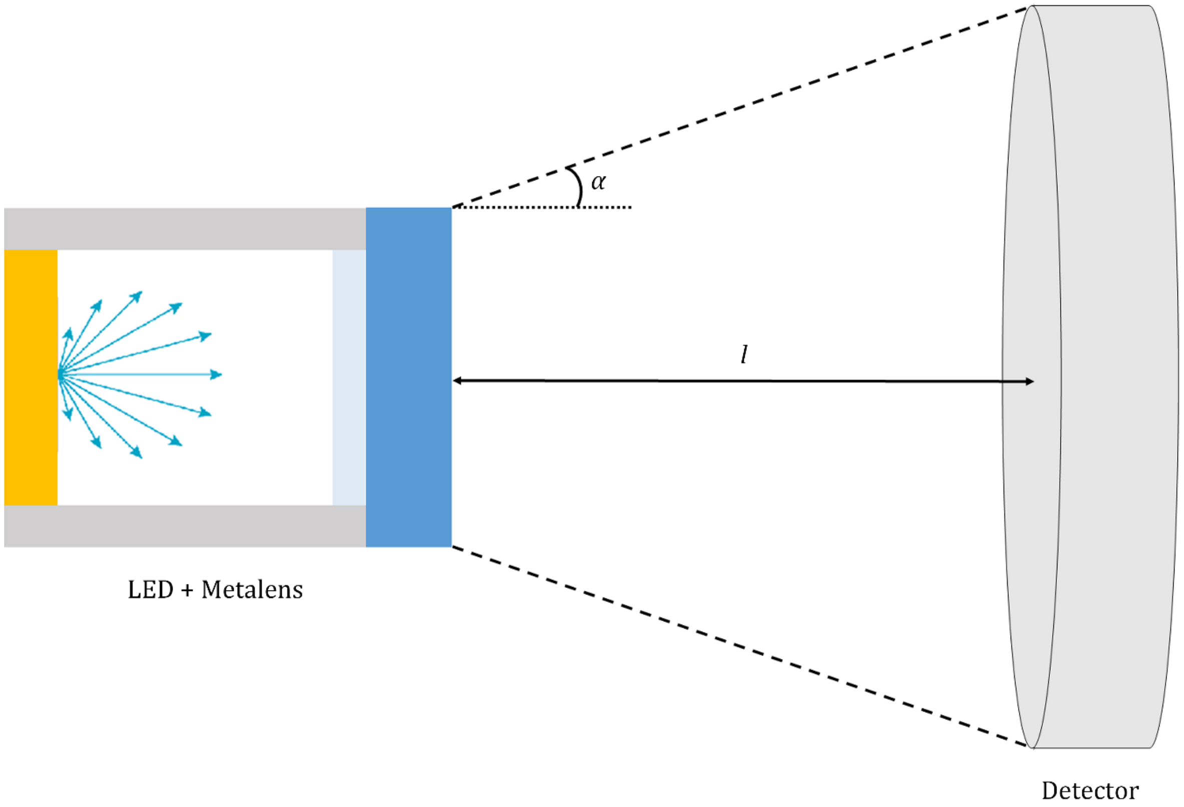

Figure S9. Measurement of the intensity within a detection angle of α. $\alpha = 0°$ or $10°$ and $l = 25$ m.

Comparison of performances

The performances of the MLs are shown in table S1 and compared with the previously reported data. The enhancements of the intensity using the MLs are numerically calculated, and the cases are suggested as ideal situations. Thus, the performances of the MLs are relatively higher than those of the others. In addition, the enhancement within the detection angle of 10° is not reported.

Table S1. Comparison of enhancement of intensity from LED sources of the previously reported results.

Method	Enhancement at α = 0°	Enhancement within α = 10°	Reference
Microlens array	219%		[1]
Surface roughening	159%		[2]
Patterned substrate	52%		[3]
Surface plasmon	330%		[4]
Surface plasmon	1,300%		[5]
Surface plasmon	1,000%		[6]
TiO_2 metalens with φ_t	8,551%	234%	This work
TiO_2 metalens with φ_m	4,973%	263%	This work
a-Si metalens with φ_t	2,115%	5%	This work
a-Si metalens with φ_m	2,013%	30%	This work

References

1. Ee, Y.K.; Arif, R.A.; Tansu, N.; Kumnorkaew, P.; Gilchrist, J.F. Enhancement of light extraction efficiency of InGaN quantum wells light emitting diodes using SiO2/polystyrene microlens arrays. *Appl. Phys. Lett.* **2007**, *91*, 221107, doi:10.1063/1.2816891.

2. An, H.M.; Sim, J.I.; Shin, K.S.; Sung, Y.M.; Kim, T.G. Increased light extraction from vertical GaN light-emitting diodes with ordered, cone-shaped deep-pillar nanostructures. *IEEE J. Quantum Electron.* **2012**, *48*, 891–896, doi:10.1109/JQE.2012.2190587.

3. Chiu, C.H.; Yen, H.H.; Chao, C.L.; Li, Z.Y.; Yu, P.; Kuo, H.C.; Lu, T.C.; Wang, S.C.; Lau, K.M.; Cheng, S.J. Nanoscale epitaxial lateral overgrowth of GaN-based light-emitting diodes on a SiO2 nanorod-array patterned sapphire template. *Appl. Phys. Lett.* **2008**, *93*, 081108, doi:10.1063/1.2969062.

4. Yu, Z.-G.; Zhao, L.-X.; Wei, X.-C.; Sun, X.-J.; An, P.-B.; Zhu, S.-C.; Liu, L.; Tian, L.-X.; Zhang, F.; Lu, H.-X.; et al. Surface plasmon-enhanced nanoporous GaN-based green light-emitting diodes with Al2O3 passivation layer. *Opt. Express* **2014**, *22*, A1596, doi:10.1364/oe.22.0a1596.

5. Okamoto, K.; Niki, I.; Shvartser, A.; Narukawa, Y.; Mukai, T.; Scherer, A. Surface-plasmon-enhanced light emitters based on InGaN quantum wells. *Nat. Mater.* **2004**, *3*, 601–605, doi:10.1038/nmat1198.

6. Zhang, H.; Zhu, J.; Zhu, Z.; Jin, Y.; Li, Q.; Jin, G. Surface-plasmon-enhanced GaN-LED based on a multilayered M-shaped nano-grating. *Opt. Express* **2013**, *21*, 13492–13501, doi:10.1364/oe.21.013492.
